# Biodegradable polymer everolimus-eluting stents versus contemporary drug-eluting stents: a systematic review and meta‑analysis

**DOI:** 10.1038/s41598-022-26654-5

**Published:** 2023-01-31

**Authors:** Juntao Yin, Yang Li, Yangyang Chen, Chaoyang Wang, Xiaoyong Song

**Affiliations:** 1 Department of Pharmacy, Huaihe Hospital, Henan University, Kaifeng, China; 2 Department of Cardiology, Huaihe Hospital, Henan University, Kaifeng, Henan China; 3 Department of General Surgery, Huaihe Hospital, Henan University, Kaifeng, Henan China; 4 Evidence-Based Medicine Center, Department of Medicine, Henan University, Kaifeng, 475000 Henan China; 5 Department of Pharmaceutics, School of Pharmacy, Henan University, Kaifeng, 475000 Henan China

**Keywords:** Cardiology, Medical research, Risk factors

## Abstract

In spite of similar efficacy and safety in pilot studies, compared with the contemporary durable polymer drug-eluting stent (DP-DES), the bioabsorbable polymer drug-eluting stent (BP-DES) may be more superior in promoting blood vessel healing. We sought to compare the safety and efficacy of everolimus-eluting BP-DES (BP-EES) with contemporary DP-DES through a meta-analysis. We performed this meta-analysis to provide further evidence of the safety and efficacy of BP-EES. Medline, Embase and the Cochrane library databases were searched for randomized controlled trials comparing clinical efficacy and safety of BP-EES versus contemporary DP-DES. Fifteen RCTs with a total of 15,572 patients were selected. The rate of MACE was 9.4% in patients receiving BP-EES and 7.3% receiving DP-EES (RR 1.13, 95% CI 0.99–1.29, *p* = 0.05; *I*^2^ = 46%). TLF and MI were also similar in both groups. Based on the available data, this review demonstrates that BP-EES displays a clinically comparable efficacy and safety profile to that of contemporary DP-DES at years of follow-up in patients undergoing PCI.

## Introduction

Implantation of drug-eluting stent (DES) that consists of a metal platform and a polymer coating with controlled release of antiproliferative agent has become the standard approach for percutaneous coronary intervention (PCI)^1,2^. Although the DES implantation could reduce the rate of restenosis, the lifelong presence of a durable polymer (DP) in a coronary artery could result in persistent arterial inflammation, delayed vessel healing, and occasionally increases the risk of severe complications such as stent thrombosis (ST), very late stent thrombosis (VLST), myocardial infarction (MI)^3^ and in-stent restenosis (ISR)^4^. These shortcomings had driven stent iterations incorporating DES with biodegradable coatings (BP-DES) that leave only bare metal scaffolds after polymer resorption, and raised the obvious question of whether the development of BP-DES will improve outcomes. Compared with contemporary DP-DES, BP-DES have comparable clinical outcomes^5,6^. The potential influence of other factors on the outcomes, such as polymer coatings composition and scaffolds strut thickness^7^, has been the focus of controversy^8,9^. It is worth noting that the strut thickness of existing BP-DES varies significantly, which may be partly the cause that BP-DES fails to show superiority over DP-DES^10,11^. Because thinner stent struts could reduce the incidence of in-stent restenosis (ISR) and target lesion revascularization (TLR)^12^, Nowadays, novel BP-DES have uncoated struts and the struts are only half as thick as that of the contemporary BP-DES^3^. BP-EES, a novel thin-strut (74–79 μm) platinum chromium alloy stent, delivers abluminal everolimus from an ultrathin poly-lactide-co-glycide (PLGA) biodegradable polymer^13^.

Results from several recently published randomized controlled trials (RCTs) are inspiring, showing that PCI with BP-EES or DP-DES is of similar outcomes^14,15^. In this meta-analysis of RCTs, we made efforts to assess the clinical efficacy and safety of BP-EES versus contemporary DP-DES.

## Methods

This study was conducted according to the Preferred Reporting Items for Systematic Reviews and Meta-Analysis (PRISMA) guidelines^16^**.**

### Search strategy

A systematic literature search was conducted by two authors independently using MEDLINE, Embase, and the Cochrane Library from inception to January 15, 2022 for RCTs comparing BP-EES with latest generation DP-DES. The search keywords were as follows:“everolimus” AND “stent” AND “biodegradable polymer” OR “bioresorbable polymer” OR “bioabsorbable polymer”.

### Eligibility criteria

Eligibility criteria were developed in accordance with the PICOS approach^17^. To perform this meta-analysis, in which all enrolled RCTs had been published, we used the PRISMA 2020 27-item checklist^18^. No restrictions on language, year, or study design were imposed, and the search strategy complied with the PRESS Guidelines^19^. Retrieve and carefully manual search the list of references for the original paper to identify other relevant studies.

### Data extraction and quality assessment

Two investigators (JY and CW) independently reviewed the eligibility of retrieved articles. Discrepancy will be resolved through consultation with a third independent investigator (XS). Pre-specified data were extracted from each enrolled study including: study design and duration, demographic and clinical characteristics of the study population, and duration of follow-up. Outcomes of interest including cardiac death, ST, MI, TLR, TLF, all-cause mortality, vessel restenosis, and MACE, were extracted as counts and percentages and recorded according the intention-to-treat (ITT) principle.

Two authors (JY and XS) independently assessed risk of bias (ROB) using the Cochrane risk of bias tool version 2.0 for RCTs^20^. All conflicts were resolved through discussion. The Robvis web application was used to produce and visualize relevant plots^21^. Authors JY and CW conducted the Grading of Recommendations, Assessment, Development and Evaluations (GRADE) independently via the GRADEpro GDT web application and resolved conflicts through discussion^22^.

### Data synthesis and analysis

Baseline risk factors and outcomes were reported as pooled proportions or mean differences (MD) with 95% confidence intervals (CI). Random effect model was selected to calculate the pooling relative risk (RR) and its 95% CI^23^. If no statistical heterogeneity was present, a fixed-effect model was used to pool data. However, if there was heterogeneity (*I*^2^ ranging from 50 to 75%), a random-effects model was used to pool data.

Heterogeneity among trials was evaluated using the *I*^2^ statistic and the Chi^2^ test^24^. *I*^2^ values of < 25%, 25–50% and > 50% correspond to low, moderate and high levels of heterogeneity, respectively. For the Chi^2^ test, a *P* value of 0.10 was considered statistically significant. We investigated potential explanations for heterogeneity by visually inspecting the forest plot. Funnel plots were used to calculate publication bias when two or more trials were enrolled. Statistical significance for hypothesis test was set at the level of 0.05. The Review Manager 5.4 (Copenhagen: The Nordic Cochrane Centre, The Cochrane Collaboration, 2020) was used to synthesize the data on an ITT basis.

## Results

### Study selection and characteristics

**Figure 1:**
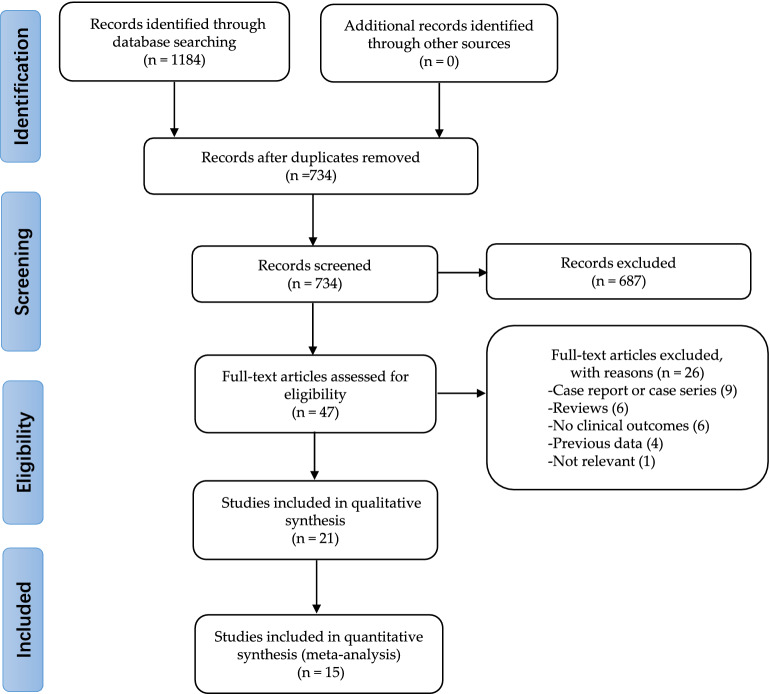
The flowchart of the database search and study selection.

A PRISMA flowchart illustrates the selection process and the number of articles, with reasons included for why studies were excluded at each step of the meta-analysis 47 (Fig. 1). Fifteen RCTs met our inclusion criteria^2,13,25–37^. Some data are from shorter follow-up time of the same study^25,31^ or from a single center in a multi-center study^13,36–38^. Therefore, a total of ten RCTs with 15,572 patients were included in the final analysis. Among these patients, 5290 were randomized to receive a BP-EES, and 10,282 patients to receive a DP-DES (DP-EES (n = 2440) and DP-zotarolimus eluting stent (ZES) (n = 1438). Unfortunately, we cannot determine how many of the other 6404 people in a study received either of the two DP-DES)^27^. Characteristics of included trials and stent used are presented in Table 1.

**Table 1:** Characteristics of the included trials and stent used.

Study/first author	Number of patients (N)		DAPT Duration (months)	Follow-up (months)	BP-EES characteristics					DP-DES characteristics			
	BP-EES	DP-DES			Stent	Strut Thickness (μm)	Scaffoldmaterial	Drug release (months)	Polymer biodegradation (months)	Stent	Strut thickness (μm)	Drug	Scaffold material
Baber 2020	491	4990	> 12	15	Synergy	74	Pl-Cr	3	4	Xience V Resolute Integrity Resolute Onyx Promus Element	81 91 81 or 90 81	Everolimus zotarolimus zotarolimus Everolimus	Co-Cr Co-Cr Co-Cr Pl-Cr
Birgelen 2016	1172	1173	> 6	12	Synergy	74	Pl-Cr	3	4	Resolute Integrity	91	zotarolimus	Co-Cr
Buiten 2020	252	265	> 6	24	Synergy	74	Pl-Cr	3	4	Resolute Integrity	91	zotarolimus	Co-Cr
Chevalier 2016	335	166	> 6	24	Absorb	150	PLLA and PDLLA	24	36	Xience V	81	Everolimus	Co-Cr
Han 2017	205	207	> 6	12	Synergy	74	Pl-Cr	3	4	Promus Element	81	Everolimus	Pl-Cr
Kereiakes 2017	1322	686	> 12	36	Absorb	150	PLLA and PDLLA	24	36	Xience V	81	Everolimus	Co-Cr
Kereiakes 2019	846	838	> 12	60	Synergy	74	Pl-Cr	3	4	Promus Element	81	Everolimus	Pl-Cr
Meredith 2018	92	98	> 6	60	Synergy	74	Pl-Cr	3	4	Promus Element	81	Everolimus	Pl-Cr
Onuma 2016	261	130	> 12	24	Absorb	150	PLLA and PDLLA	24	36	Xience V	81	Everolimus	Co-Cr
Xu 2018	236	235	> 12	36	Absorb	150	PLLA and PDLLA	24	36	Xience V	81	Everolimus	Co-Cr

*BP-EES* biodegradable polymer everolimus-eluting stent(s), *Co-Cr* cobalt-chromium, *DAPT* dual-antiplatelet therapy, *DP-DES* durable polymer drug-eluting stent(s), *PDLLA*, poly(D,L-lactide), *Pl-Cr* latinumchromium, *PLLA* poly(L-lactide), *SS* stainless steel.

Patients characteristics on the included trials are presented in Table 2. No difference was found in age (pooled mean, 61.2 vs. 61.4 years, *p* = 0.69), male sex (73.8 vs. 72.2%, *p* = 0.42), smoking habit (28.9 vs. 30.5%, *p* = 0.30), diabetes (26.7 vs. 26.8%, *p* = 0.78), hypertension (66.0 vs. 67.3%, *p* = 0.96), dyslipidaemia (59.3 vs. 60.0%, *p* = 0.46), or unstable angina (26.6 vs. 27.0%, *p* = 0.74). Patients who received DP-DES had a higher prevalence of prior MI (18.6 vs. 21.9%, *p* < 0.001) compared with BP-EES. Table 2 also present procedural characteristics and there was no difference among treated vessels.

**Table 2:** Patients and procedural characteristics.

Baseline characteristics	No. of studies	BP-EES	DP-DES	*p* value	*I*^2^	Fixed-effects estimates
Age, years	10	61.2 ± 10.3	61.4 ± 10.2	0.69	38%	0.83 [0.62, 1.06]
Male	10	73.8 ± 4.35	72.2 ± 3.3	0.42	47%	1.02 [0.98, 1.06]
Smoking habit	8	28.9 ± 14	30.5 ± 14	0.30	0%	0.97 [0.92, 1.03]
Diabetes	10	26.7 ± 7.7	26.8 ± 6.9	0.78	0%	1.01 [0.95, 1.07]
Hypertension	10	66.0 ± 13.5	67.3 ± 12.3	0.96	23%	1.00 [0.97, 1.03]
Dyslipidaemia	9	59.3 ± 22.4	60.0 ± 23.3	0.46	0%	0.99 [0.96, 1.02]
Prior myocardial infarction	10	18.6 ± 7.6	21.9 ± 8.8	< 0.0001	2%	0.85 [0.79, 0.92]
Unstable angina	9	26.6 ± 17.4	27.0 ± 16.8	0.74	14%	0.99 [0.91, 1.07]
**Treated vessels**						
LAD	10	46.8 ± 5.8	45.5 ± 6.4	0.42	0%	1.02 [0.98, 1.06]
Cx	9	24.5 ± 5.2	26.8 ± 3.9	0.07	21%	0.93 [0.87, 1.01]
RCA	10	33.6 ± 6.6	32.1 ± 5.4	0.19	3%	1.03 [0.98, 1.08]
Left main	4	3.8 ± 4.2	4.2 ± 4.6	0.28	0%	0.86 [0.65, 1.13]
Reference vessel diameter, mm	6	2.7 ± 0.1	2.7 ± 0.1	0.54	0%	0.76 [0.62, 1.19]
Minimal lumen diameter, mm	6	0.8 ± 0.1	0.8 ± 0.1	0.92	48%	1.02[0.84, 1.21]
Total lesion length, mm	5	14.8 ± 1.2	14.6 ± 1.1	0.34	32%	0.95[0.80, 1.17]
Stenosis diameter	5	70.8 ± 5.9	70.2 ± 5.4	0.08	39%	0.79 [0.58, 1.03]
Stent length, mm	4	26.9 ± 8.9	26.9 ± 9.1	0.16	20%	0.97 [0.78, 1.05]

Bold values showing statistical significant difference. Values are proportions, mean differences or RR with 95% confidence intervals (in parentheses).

*BP-EES* biodegradable polymer everolimus-eluting stent, *DP-DES* durable polymer drug-eluting stent, *CI* confidence interval, *CABG* coronary artery bypass grafting, *PCI* percutaneous coronary intervention, *LAD* left anterior descending artery, *Cx* circumflex artery, *RCA* right coronary artery.

**Figure 2:**
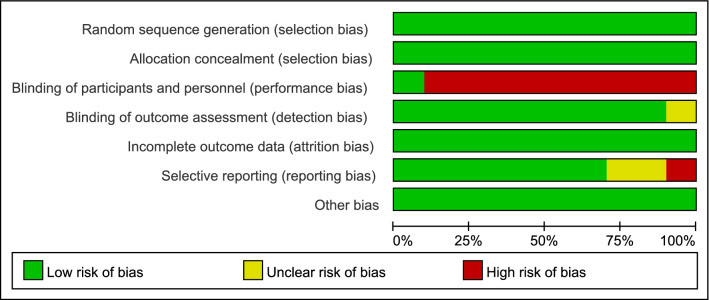
Risk of bias summary: review authors’ judgements about each risk of bias item for each included study.

**Figure 3:**
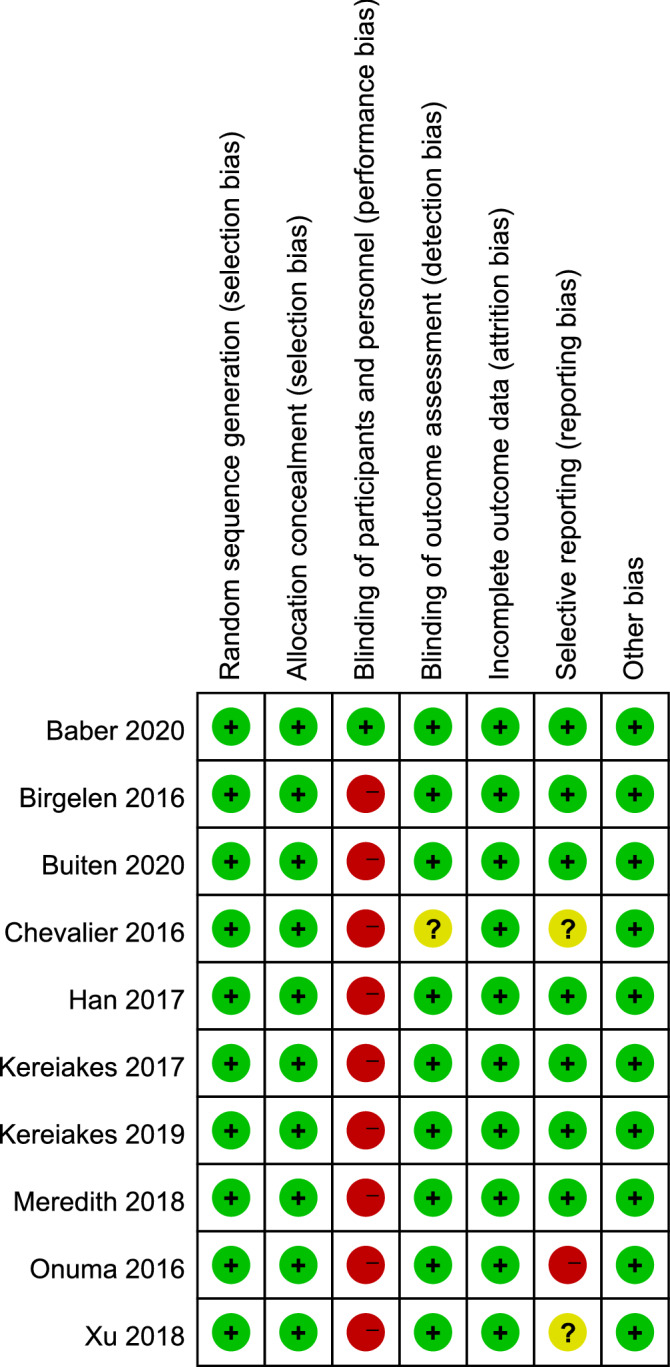
Risk of bias graph: review authors' judgements about each risk of bias item presented as percentages across all included studies.

Risk of bias was predominately low across all studies except in regards to performance bias (Fig. 2). The individual items of the risk of bias are presented in Fig. 3.

### Primary outcome TLF

**Figure 4:**
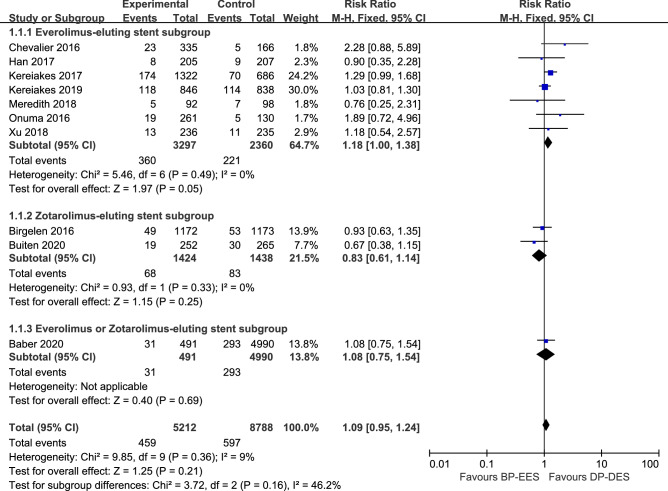
The pooled RR of target-lesion failure between patients intervened with BP-EES and DP-DES with subgroup analysis.

**Figure 5:**
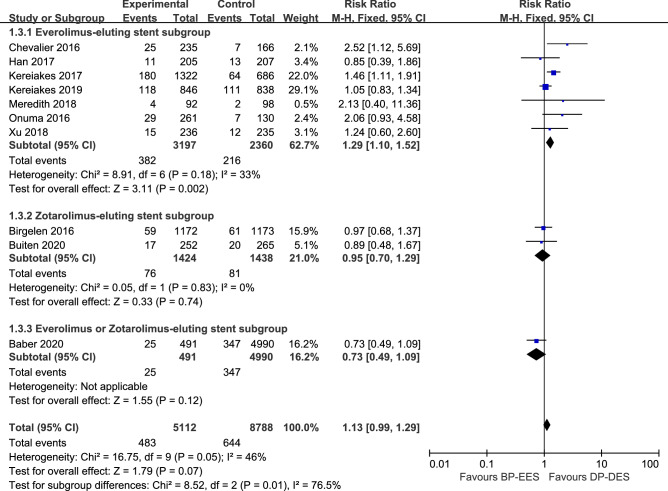
The pooled RR of major adverse cardiovascular event between patients intervened with BP-EES and DP-DES with subgroup analysis.

**Figure 6:**
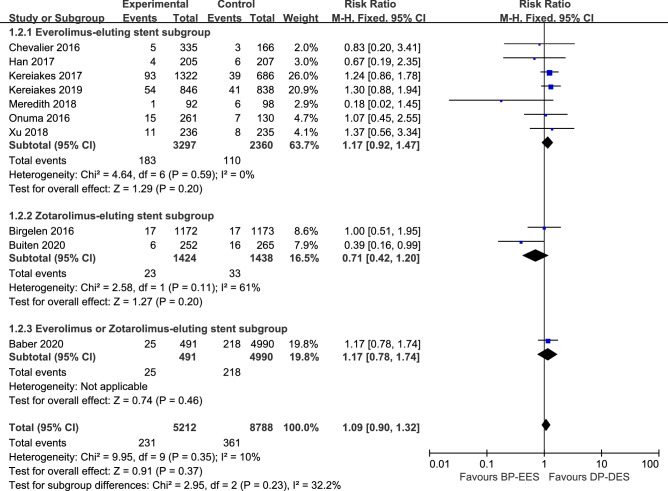
The pooled RR of target lesion revascularization between patients intervened with BP-EES and DP-DES with subgroup analysis.

Target-lesion failure (TLF), a composite endpoint of cardiac death, MI, and clinically indicated TLR, represent a safety and efficacy outcome^38^. Study-level outcomes at longest available follow-up for MACE, the individual components of MACE and TLR are summarized in Table 3. TLF was comparable between patients intervened with BP-EES and DP-DES (8.8% vs. 6.8%; RR 1.09, 95% CI 0.95–1.24; *I*^2^ = 9%) (Fig. 4).The rate of MACE was 9.4% in patients receiving BP-EES and 7.3% receiving DP-EES (RR 1.13, 95% CI 0.99–1.29; *I*^2^ = 46%) (Fig. 5). The rate of TLR was also comparable between patients intervened with BP-EES and DP-DES (4.4% vs. 4.1%, RR 1.09, 95% CI 0.90–1.32; *I*^2^ = 10%) (Fig. 6).

**Table 3:** Cardiac outcomes.

Analysis (no. of trials included)	TVR RR (95% CI)	Cardiac death RR (95% CI)	MI RR (95% CI)	Definite/probable ST RR (95% CI)
Outcomes at 1 year (2)	0.72 [0.45, 1.17]	1.00 [0.42, 2.40]	0.88 [0.54, 1.44]	0.73 [0.25, 2.20]
Outcomes at the longest follow-up (8)	0.95 [0.64, 1.41]	0.79 [0.55, 1.15]	1.18 [0.99, 1.42]	1.63 [1.01, 2.64]
Landmark analysis beyond 1 year (8)	0.95 [0.64, 1.41]	0.79 [0.55, 1.15]	1.18 [0.99, 1.42]	1.63 [1.01, 2.64]
**Subgroup analysis**				
**BP-EES strut thickness**				
Thin struts (6)	0.64 [0.39, 1.06]	0.76 [0.51, 1.12]	1.03 [0.83, 1.28]	0.80 [0.45, 1.43]
Thick struts (4)	1.40 [1.08, 1.82]	1.07 [0.53, 2.19]	1.34 [1.01, 1.77]	3.07 [1.46, 6.46]
**DP eluting drug**				
Everolimus (7)	1.10 [0.81, 1.48]	0.82 [0.56, 1.21]	1.19 [0.99, 1.44]	1.66 [1.01, 2.72]
Zotarolimus (2)	0.53 [0.22, 1.25]	0.83 [0.40, 1.71]	0.89 [0.58, 1.38]	0.79 [0.30, 2.12]
**DAPT duration**				
≥ 6 months (5)	0.53 [0.33, 0.85]	0.96 [0.49, 1.89]	0.94 [0.64, 1.38]	1.01 [0.43, 2.36]
≥ 12 months (5)	1.24 [1.03, 1.50]	0.78 [0.53, 1.16]	1.20 [0.99, 1.45]	1.64 [0.98, 2.74]

*MI* myocardial infarction, *RR* risk ratio, *ST* stent thrombosis, *TVR* target vessel revascularization, other abbreviations as in Table 1.

### Secondary outcomes MI, cardiac death and ST

The meta-analysis showed no significant difference between BP-EES and DP-DES in MI (5.9% vs. 4.4%; RR 1.14, 95% CI 0.96–1.35; *I*^2^ = 0%) (Fig. S1), ST (1.2% vs. 0.8%; RR 1.63, 95% CI 1.00–2.66; *I*^2^ = 7%) (Fig. S2), cardiac mortality (1.3% vs. 1.4%, RR 0.82, 95% CI 0.58–1.15; *I*^2^ = 0%) (Fig. S3), All-cause death (2.8% vs. 2.3%, RR 0.94, 95% CI 0.74–1.18; *I*^2^ = 0%) (Fig. S4), or Target vessel failure (11.5% vs. 4.1%, RR 1.26, 95% CI 0.51–3.12; *I*^2^ = 98%) (Fig. S5). There was no significant difference between BP-EES and DP-DES in Target vessel revascularization (7.3% vs. 6.4%, RR 0.89, 95% CI 0.64–1.25; *I*^2^ = 62%) (Fig. S6). Again, no significant difference was noted between BP-EES and DP-DES in non-target lesion revascularization target vessel revascularization (non-TLR TVR) (2.7% vs. 2.6%, RR 0.77, 95% CI 0.38–1.56; *I*^2^ = 65%) (Fig. S7).

## Discussion

Prior meta-analysis^39^ demonstrated no significant difference in clinical outcomes at one-year follow-up in patients treated with BP-EES (only include the *Synergy*™ stent) or DP-DES. This current meta-analysis is the largest sample size meta-analysis comparing BP-EES to contemporary DP-DES, which involves 15 RCTs with 15,772 patients’ years of follow-up. In our meta-analysis, we demonstrated that both BP-EES and contemporary DP-DES had similar safety and efficacy profiles in patients with obstructive coronary artery disease. Although there was numerical increase in TVR and reduction in cardiac mortality with BP-EES, there was no statistically significant difference, with regard to the low rates in both groups. There was no significant difference in TLR, MI, ST or TLF, MACE when comparing BP-EES with contemporary DP-DES. In addition to BP-EES, the biolimus-eluting *Nobori* stent and bioresorbable Sirolimus-eluting *MiStent*, have shown similar difference in TLR and ST compared with contemporary DP-DES^40,41^.

Interestingly, while there was a trend for less TVR associated with BP-EES in several studies^2,13,28,29^, this meta-analysis of all available RCTs showed no significant difference between BP-EES and contemporary DP-DES. The outcomes, while demonstrating no superiority of BP-EES, suggest that the BP-EES is not inferior to newer, widespread used DP-DES. Furthermore, considering ST with the *Absorb*™ (*Abbott Vascular*) bioresorbable vascular scaffold^42,43^, thses data do not cause BP-EES safety concerns. Current generation DP-DES with superior antiproliferative drug, pharmacokinetic release profile, stent, and strut thickness, demonstrating better efficacy compared with previous generation stents^11,44^, remains the benchmark for comparison. However, durable polymer coating in these stents might lead to chronic arterial inflammation and incomplete endothelization, resulting in delayed vascular healing and nidi for ST and VLST^45^. Indeed, whether BP-DES can improve clinical efficacy when compared with newer DP-DES has been a controversial topic^46^ and may be affected by other factors, such as polymer composition and strut thickness^7^. Some research has further developed approaches to meet challenges in the design of polymeric drug delivery systems^47^. It is obvious that there is significant variability in the strut thickness of available BP-DES, which may account for the efficacy inconsistency between BP-DES and DP-DES^48,49^. This inconsistency maybe also be associated with the biocompatible polymer of contemporary DP-DES and subsequent improvements in safety and efficacy, which counteract the benefits of BP^7^. Newer generation ultra-thin strut BP-DES (strut thickness < 70 μm) could cause a 16% reduction in incidence of TLF driven by lower rates of MI and ST^12,50^. Additionally, the advantages of thin or ultra-thin stents BP-DES, which can reduce platelet aggregation and inflammatory cell adhesion^51,52^, may be very helpful in certain clinical situations, such as small-vessel PCI or in-stent restenosis.

The current meta-analysis cannot show a significantly reduced risk of late ST/VLST between BP-DES and DP-DES. Eight of the ten trials included in the study (including two 5-years and two 3-years) present a follow-up for more than two-year post implantation. Previous RCTs and meta-analyses demonstrated that BP-DES were associated with lower incidence of late ST/VLST compared with either bare metal stents or first generation DES^53^. Furthermore, recent studies have shown lower rates of ST in the newer generation cobalt chromium (CoCr) and PtCr durable polymer (polyvinylidene fluoride) EES than other DP-DES, early BP-DES and bare metal scaffolds^54,55^. Eventually, a large sample size RCT of the CoCr EES vs. the *Nobori*™ (*Terumo*) BP-DES showed comparable long-term outcomes between both stents^56^. These obvious discrepancies may be partly due to the design difference of BP-DES platform^6^. Many factors, such as metal alloys, stent strut thickness, polymer composition, and distribution, may affect the time course of biodegradable polymer and scope of endothelial stent coverage, as well as the function and maturation of endothelial cells^7,57^. These aspects emphasize that device specific analysis is more important than stent category.

This paper, like any meta-analysis, also shares the limitations of original research. First, we can access no information on the duration of DAPT, which may influence the clinical outcomes. Second, this is a study-level meta-analysis, so it is impossible to investigate the role of several confounders at the patient level. Third, we grouped all types of DP-DES into a control group, which may cause potential heterogeneity. However, our subgroup analysis has no significant interaction between the two different types of device. The small number of DP-ZES subgroup studies may limit results of subgroup analysis, and reliability of the conclusions may be decreased by moderate heterogeneity of some secondary outcome analysis.

Since we included only RCTs and used all available study data, the likelihood of publication bias seems low. Although this meta-analysis included 15 RCTs with 15,772 patients, it may still be insufficient to assess minor difference in the incidence of rare adverse events such as ST/VLST.

## Conclusions

In this meta-analysis comparing BP-EES with contemporary DP-DES, no significant differences in clinical outcomes were found between the two platforms, which suggests that the safety and efficacy of BP-EES are comparable to contemporary DP-DES.

## Supplementary Information

Supplementary Information 1.

Supplementary Information 2.

## Data Availability

The present study is a meta-analysis of published randomized trials. All data used for analyses are presented in the manuscript.

## Abbreviations

BP-DES: Bioabsorbable polymer drug-eluting stent
BP-EES: Bioabsorbable polymer everolimus-eluting stent
CI: Confidence intervals
CoCr: Cobalt chromium
DP: Durable polymer
DP-DES: Durable polymer drug-eluting stent
ISR: In-stent restenosis
ITT: Intention-to-treat
MACE: Major adverse cardiovascular event
MD: Mean differences
MI: Myocardial infarction
non-TLR TVR: Non-target lesion revascularization target vessel revascularization
PCI: Percutaneous coronary intervention
PLGA: Poly-lactide-co-glycide
RCT: Randomized Controlled Trial
RR: Risk Ratio
ST: Stent thrombosis
TLF: Target-lesion failure
TLR: Target lesion revascularization
TVR: Target vessel revascularization
VLST: Very late stent thrombosis
ZES: Zotarolimus eluting stent
