# Supplementary material for: Biodegradable polymer everolimus-eluting stents versus contemporary drug-eluting stents: a systematic review and meta‑analysis
Source: Sci Rep. 2023 Jan 31;13:1715. doi: 10.1038/s41598-022-26654-5 (PMC9889391; doi:10.1038/s41598-022-26654-5)
Supplement: Supplementary file 2 — Supplementary Information 2. [file 41598_2022_26654_MOESM2_ESM.docx]

**Supplementary file**

**
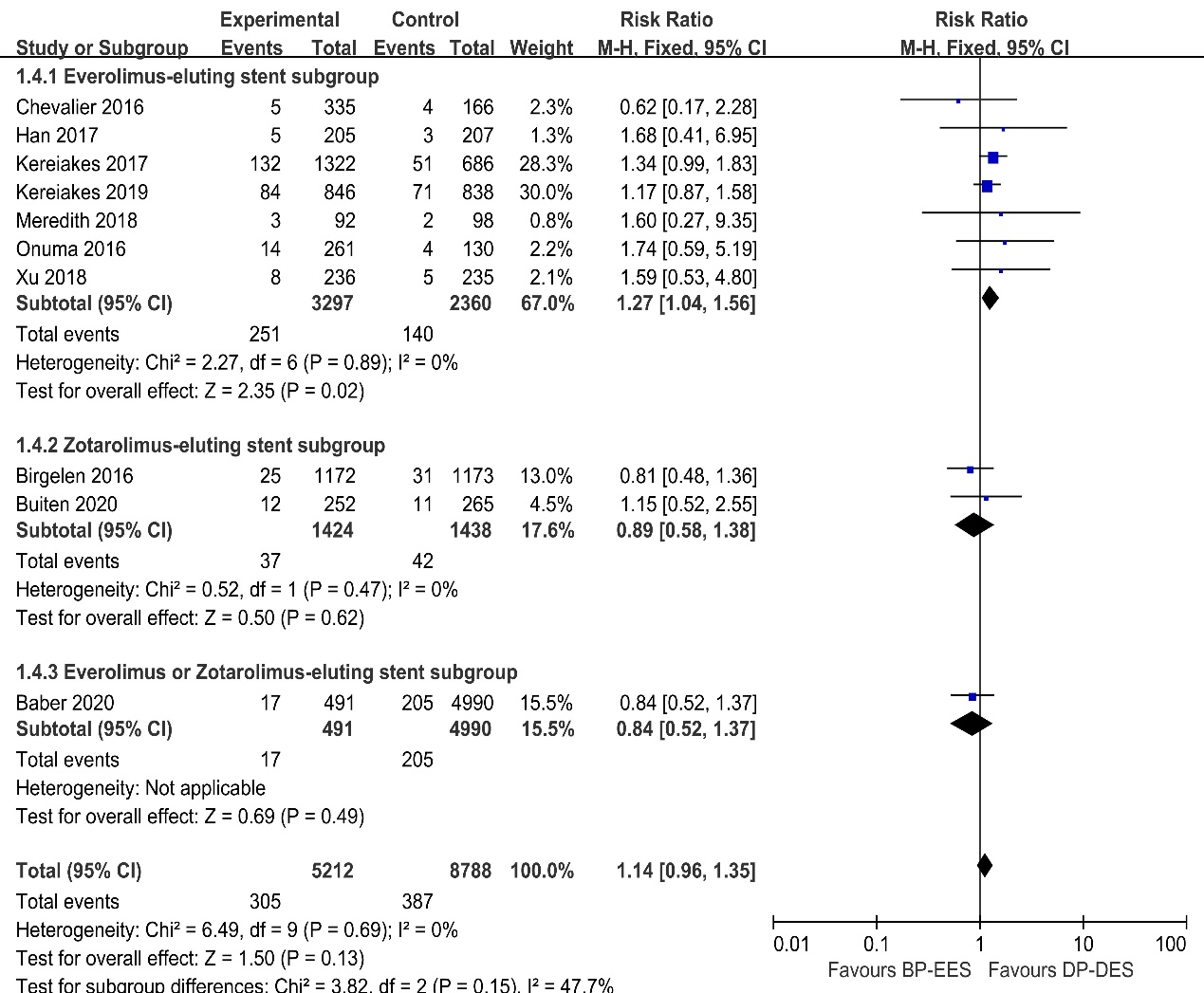
Fig.** **S1** The pooled RR of myocardial infarction between patients intervened with BP-EES and DP-DES with subgroup analysis.

**
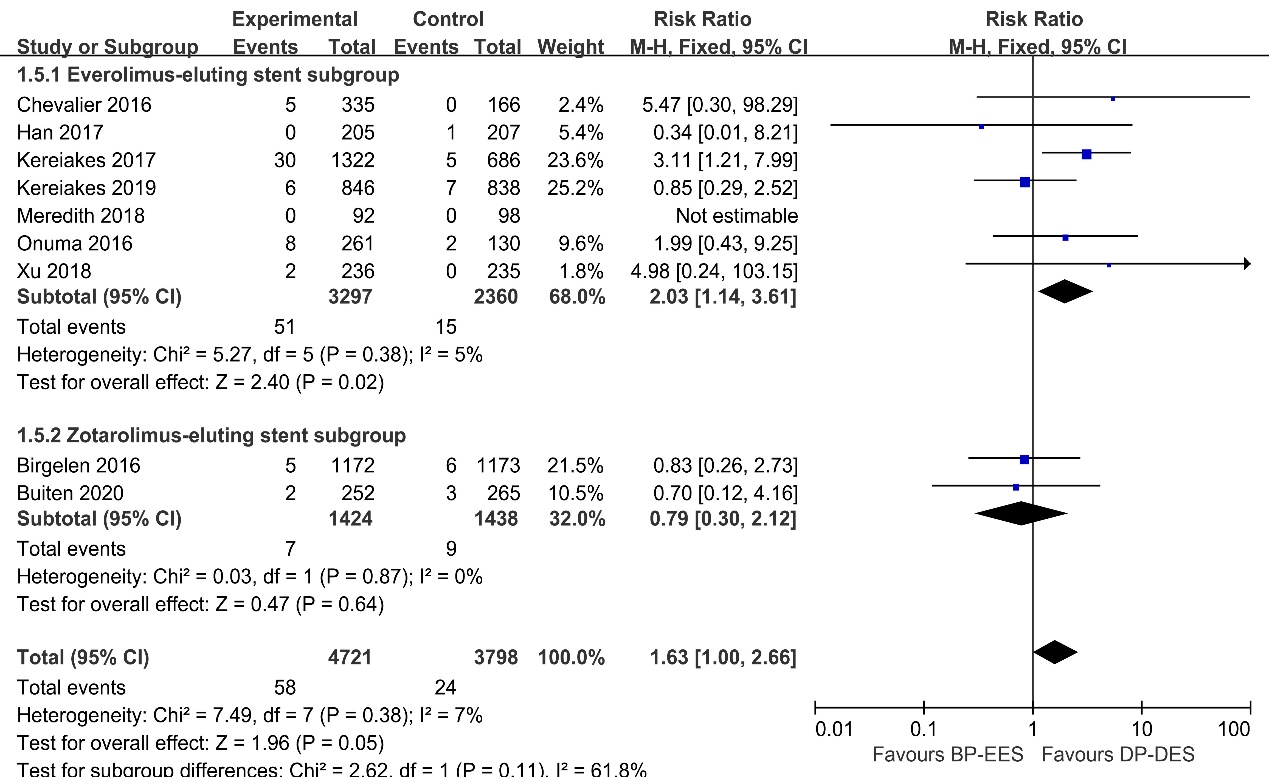
Fig.** **S2** The pooled RR of stent thrombosis between patients intervened with BP-EES and DP-DES with subgroup analysis.

**
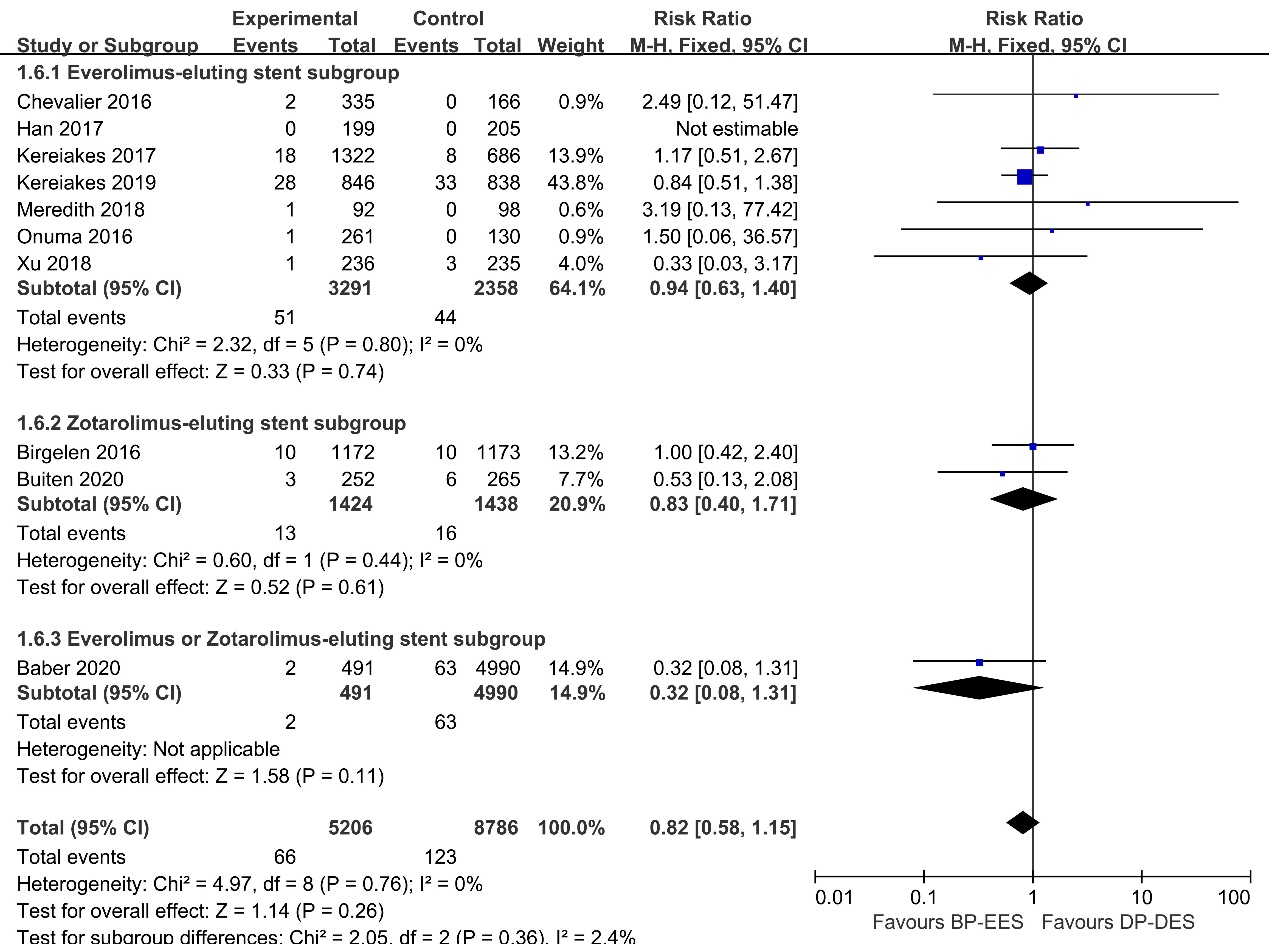
Fig.** **S3** The pooled RR of cardiac mortality between patients intervened with BP-EES and DP-DES with subgroup analysis.

**
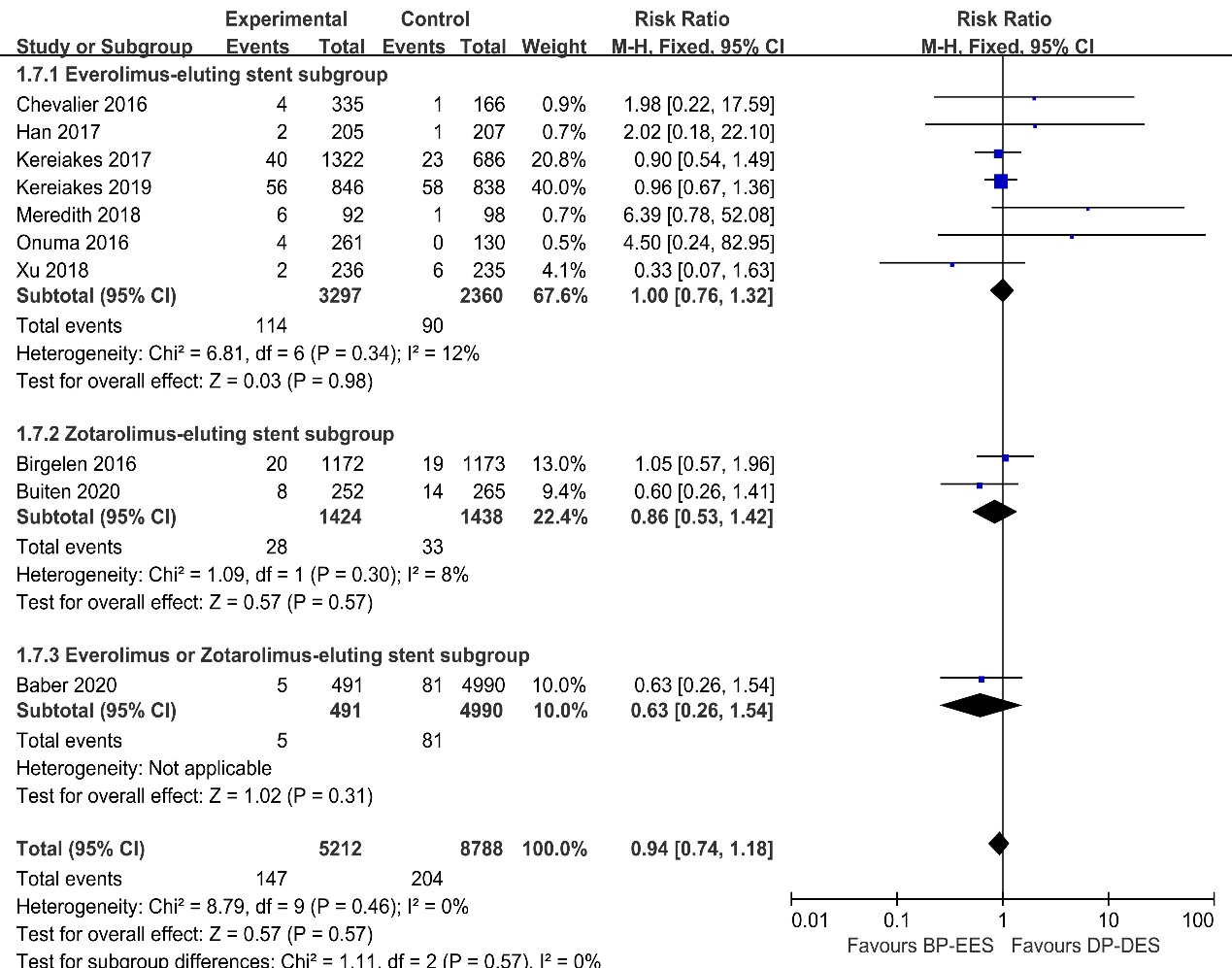
Fig.** **S4** The pooled RR of all-cause death between patients intervened with BP-EES and DP-DES with subgroup analysis.

**
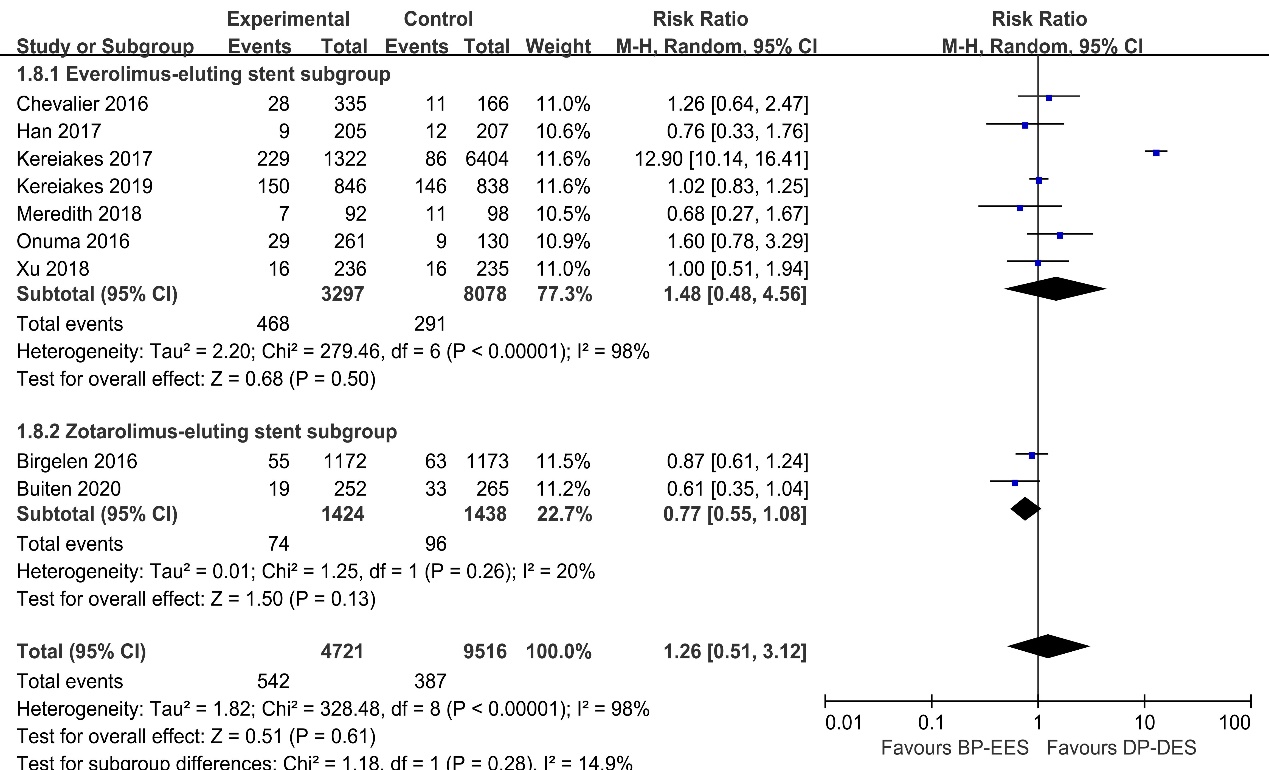
Fig.** **S5** The pooled RR of target vessel failure between patients intervened with BP-EES and DP-DES with subgroup analysis.

**
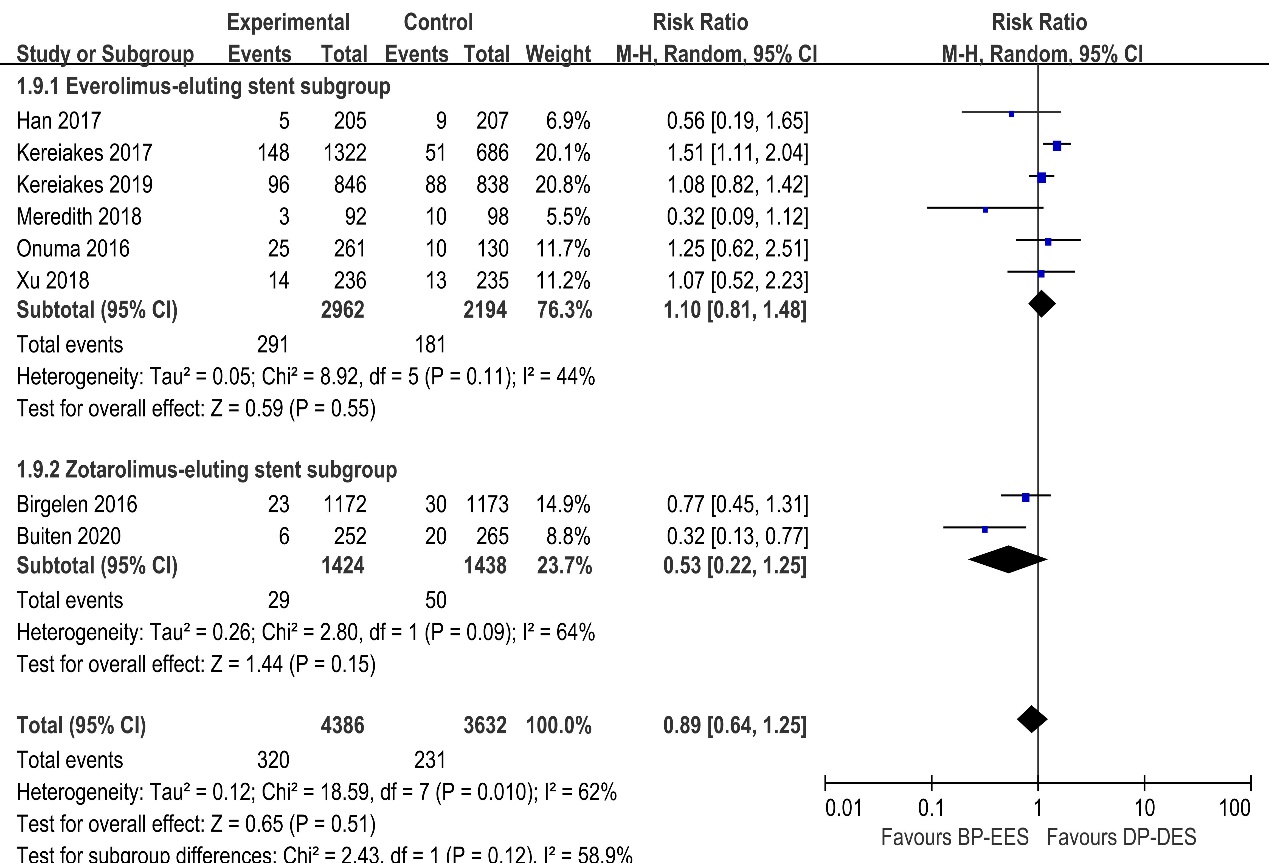
Fig.** **S6** The pooled RR of target vessel revascularization between patients intervened with BP-EES and DP-DES with subgroup analysis.

**
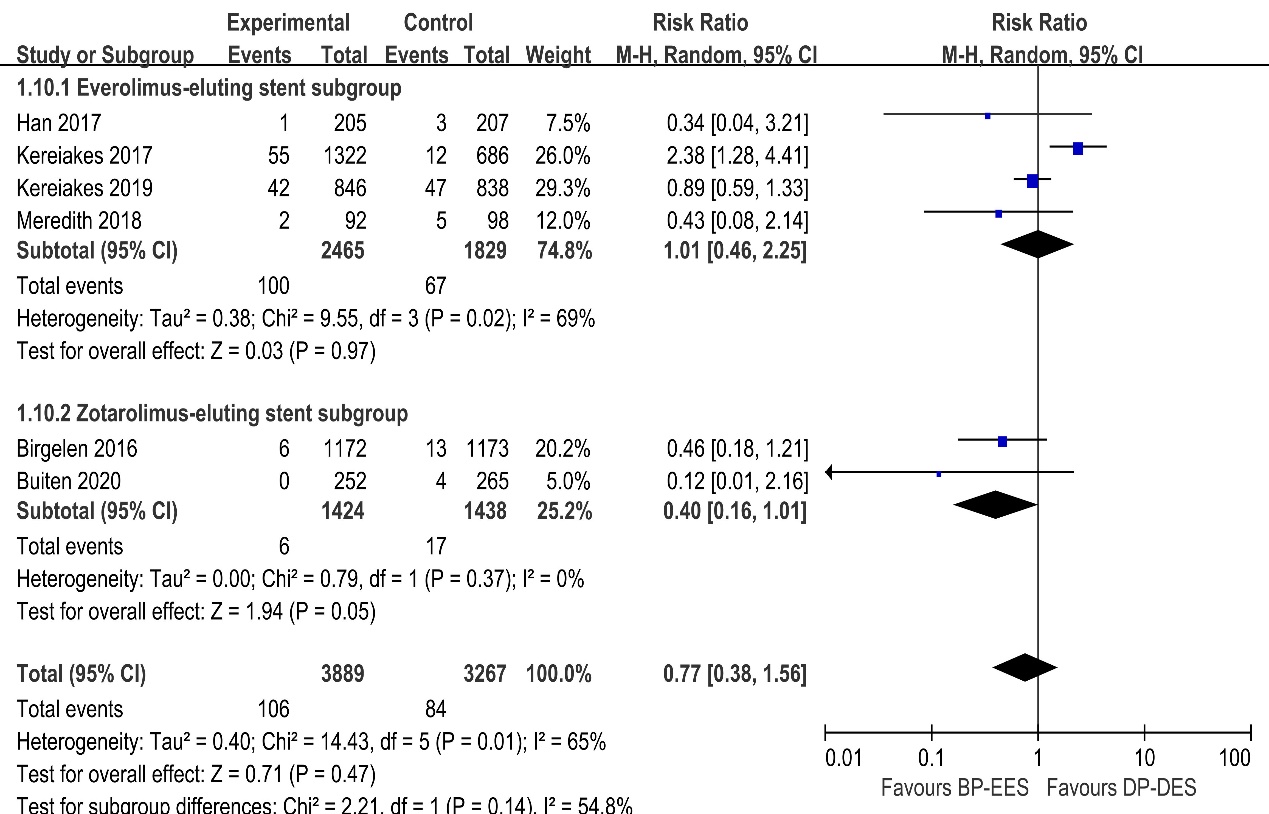
Fig.** **S7** The pooled RR of non-target lesion revascularization target vessel revascularization between patients intervened with BP-EES and DP-DES with subgroup analysis.
